# Rinsing with L-Ascorbic Acid Exhibits Concentration-Dependent Effects on Human Gingival Fibroblast In Vitro Wound Healing Behavior

**DOI:** 10.1155/2020/4706418

**Published:** 2020-03-21

**Authors:** Tatcha Chaitrakoonthong, Ruchanee Ampornaramveth, Paksinee Kamolratanakul

**Affiliations:** ^1^Oral and Maxillofacial Surgery, Faculty of Dentistry, Chulalongkorn University, Bangkok 10330, Thailand; ^2^Microbiology, Faculty of Dentistry, Chulalongkorn University, Bangkok 10330, Thailand

## Abstract

Vitamin C or L-ascorbic acid has diverse functions in the body, especially healing promotion in tissue injury via participating in the hydroxylation reactions required for collagen formation. Systemic administration of vitamin C plays an important role on gingival fibroblast proliferation and functions. Whether local or rinsing administration of vitamin C alters gingival fibroblast wound healing behavior remains unclear. The aim of this study was to investigate the rinsing effect of vitamin C on gingival fibroblast behavior utilizing an *in vitro* wound healing model. Primary human gingival fibroblasts isolated from gingival tissue were rinsed with medium containing various concentrations of vitamin C. The rinsing effect of vitamin C on in vitro wound healing was assessed using a scratch test assay. Cell migration, cell viability, and extracellular matrix gene expression were analyzed by transwell migration assay, MTT assay, and real-time RT-PCR, respectively. We found that rinsing with 10 or 20 *µ*g/ml vitamin C significantly increased fibroblast migration (*p* ≤ 0.05). However, no significant effect was found in the cell viability or in vitro wound healing assays. In contrast, rinsing with 50 *µ*g/ml vitamin C significantly delayed wound closure (*p* ≤ 0.05). Real-time PCR demonstrated that 50 *µ*g/ml vitamin C significantly increased fibroblast expression of COL1, FN, IL-6, and bFGF. The data demonstrate that rinsing with vitamin C (10/20 *µ*g/ml) accelerates fibroblast migration. However, 50 *µ*g/ml of vitamin C increases the expression of COL1, FN, IL-6, and bFGF, which are related to fibroblast wound healing activity. Prescribing vitamin C with the appropriate duration and drug administration method should be determined to maximize its benefit.

## 1. Introduction

Normal wound healing can be classified into four stages with overlapping phases: hemostasis, inflammation, proliferation, and remodeling [1–4]. Wound healing starts with a blood clot that initially seals the wound during the hemostatic phase. Platelets and inflammatory cells are the first cells to arrive and provide key functions and signals that are essential for the influx of important cells, such as fibroblasts and endothelial cells to the injury site. Within the first week, the blood clot is almost entirely replaced with granulation tissue produced by fibroblasts [1, 2, 5].

Fibroblasts play an important role in wound healing via migrating to the wound site, proliferating, and subsequently synthesizing cytokines and extracellular matrix matrix (ECM) for wound contraction and wound remodeling [5, 6]. Fibroblasts generate the ECM via the production of collagen and noncollagenous extracellular matrix proteins, e.g., fibronectin, glycosaminoglycans, and proteoglycans [7]. Fibroblast wound healing activity is regulated by various growth factors, cytokines, and chemokines, including fibroblast growth factors, interleukins (ILs), vascular endothelial growth factor (VEGF), and the transforming growth factor beta-family (TGF-*β*) [1, 8].

Vitamin C or L-ascorbic acid, a water-soluble vitamin, has various biochemical functions, including an essential role in the hydroxylation reactions that are necessary for collagen and carnitine synthesis, phagocytosis of polymorphonuclear leukocytes, differentiation of several mesenchymal cell types, and antioxidant scavenging of reactive oxygen species [9–12]. In addition, vitamin C regulates tumor growth and angiogenesis [13]. Vitamin C is an essential nutrient for humans because vitamin C cannot be synthesized endogenously in the human body [9]. Vitamin C is required for healing both soft- and hard-injured tissues [14]. Several studies have shown that vitamin C promoted fibroblast proliferation [1, 15]. Fibroblasts harvested from infants have a greater cell proliferation potential compared with those from the elderly; however, vitamin C stimulated the proliferation of both infant and elderly fibroblast cells. Vitamin C induces a dose-dependent increase in collagen type I expression by normal human fibroblasts and enhances extracellular matrix contraction [16]. Clinical studies have shown that systemic vitamin C accelerated surgical and tooth extraction socket wound healing [17–20]. However, the mechanism of how vitamin C promotes oral tissue healing or whether intermittent-local administration of vitamin C stimulates gingival fibroblasts and regulates wound healing remains unresolved. We hypothesized that rinsing or intermittent-local administration of vitamin C may show a different effect compared to systemic oral or intravenous form of vitamin C administration. Therefore, the aim of this study was to investigate whether intermittent-local exposure of human gingival fibroblasts to vitamin C promotes *in vitro* wound healing by inducing cell viability and migration and promote fibroblast wound healing activity by inducing extracellular matrix and collagen synthesis.

## 2. Materials and Methods

### 2.1. Cell Culture

Unerupted mandibular third molar gingiva specimens were obtained from healthy young volunteers (aged 18–25 years). The gingival tissue was immediately transferred in the ice-cold storage medium (DMEM (Gibco, Life Technologies Corporation, Grand Island, NY, USA) supplemented with 10% FBS, 1% L-Glutamine, 0.5 mg/ml gentamicin, and 3 mg/ml amphotericin B). Each specimen was rinsed twice in calcium- and magnesium-free Dulbecco's phosphate-buffered saline (PBS). The gingival tissues were cut into 1 × 2 mm pieces with a surgical blade and seeded in 100-mm dishes in DMEM supplemented with 10% FBS, 1% L-glutamine, and 1% antibiotics. The tissue samples were incubated at 37°C in a humidified 5% CO_2_ atmosphere, and the medium was changed every 3 days until the outgrowing cells reached confluence. The whole population of cells obtained from gingival tissues using the outgrowing technique which reassemble mainly “gingival fibroblast” was subjected to the study [21]. The primary human gingival fibroblasts (hGFs) at the 3rd–6th passage were used for the experiments. The patients provided written informed consent for the use of discarded tissue for research purposes. The study protocol was approved by the Ethics Committee of the Faculty of Dentistry, Chulalongkorn University, Thailand (HREC-DCU 2016-097). Primary hGFs from one donor were used in all experiments. The cells from three different donors were utilized to confirm the results in triplicate. Four experiments: scratch test assay, viability test, cell migration, and PCR were performed. For each experiment, one plate was used for one patient or one donor. All experimental groups (rinsing with 0, 10, 20, or 50 *µ*g/ml L-ascorbic acid) were used three wells per group. The total was 9 samples per groups in all experiments. Only data from one donor were used to demonstrate in graphs of the experimental results.

### 2.2. Scratch Test Assay (*In Vitro* Wound Healing Assay)

To evaluate the effect of L-ascorbic acid on hGF-mediated wound healing in vitro, a scratch test assay was performed. The hGFs were seeded in a 12-well plate at a density of 100,000 cells/well (26,316 cells/cm^2^). After 24 h, when the cells nearly reached confluence, a sterile 1 ml pipette tip was used to make a straight scratch line on the confluent cell monolayer. The debris was washed away with PBS. The cells were cultured at 37°C in a humidified 5% CO_2_ atmosphere. According to our rinsing protocol adapted from our previous study [21], the cells were rinsed with 0, 10, 20, or 50 *µ*g/ml L-ascorbic acid, in culture medium for 7 min, 3 times/day. At 0, 12, 24, and 48 h, the wound areas were visualized and recorded using an inverted microscope (Eclipse TS100, Nikon, Japan) and a digital camera at the same position of each culture plate using a registration mark placed on the bottom of each well. The remaining wound areas were analyzed using ImageJ 1.45S software (Wayne Rasband, National Institutes of Health, Bethesda, MD, USA) as previously described [22]. The percentage of remaining wound areas were calculated as described in the following equation.

(1) $$\begin{array}{c} \frac{\text{Area of the in vitro wound}\left(\text{scratched area}\right)\text{ at }0\text{ hour}-\text{Area remaining at }0,12,24,\text{or }48\text{ hours}}{\text{Area of the in vitro}\left(\text{scratched area}\right)\text{ at }0\text{ hour}}\times 100. \end{array}$$

### 2.3. Cell Viability Assay

Cell viability was assessed using a 0.5 mg/ml of 3-(4,5-dimethylthiazol-2-yl)-2,5-diphenyl tetrazolium bromide assay (MTT; #298-931, USB Corporation, Cleveland, OH, USA). The cells were plated at 2 × 10^4^ cells/cm^2^ in two 24-well plates. After 24 h, the medium in the wells of the 1st plate was replaced with 0.5 ml MTT solution and incubated for 30 min at 37°C. The formazan product was dissolved in solubilization/stop solution consisting of 9 : 1 DMSO and glycine buffer. The optical densities were measured using a microplate reader (ELx800, BioTek, Winooski, VT, USA) at 570 nm. The cells in the 2^nd^ plate were rinsed with 0, 10, 20, or 50 *µ*g/ml L-ascorbic acid for 7 min, 3 times/day. After 48 h, the MTT assay was performed on the cells in the 2^nd^ plate as described above. The absorbance results were normalized to day 1 results and compared between groups.

### 2.4. Transwell Migration Assay

The cell migration assay was performed using 24-well size transwell inserts with an 8.0 *µ*m pore polycarbonate membrane with a 0.3 cm^2^ effective growth area (#3422, BD Falcon™ Cell Culture Inserts, BD Biosciences, Bedford, MA, USA). The hGFs were trypsinized and resuspended in serum-free media. 1 × 10^5^ cells in 200 *µ*l of medium were seeded on each insert. One hour after seeding, the cells were rinsed with their respective group's medium. In brief, the inserts were moved to new 24-well plates containing 700 *µ*l of 0, 10, 20, or 50 *µ*g/ml L-ascorbic acid in serum-free medium for 7 min. The rinsing procedure was repeated 3 times/day. To rule out the effect from cell proliferation, the migration assay was performed with serum-free medium for 24 h. On the next day, the nonmigrated cells from the upper surface of the membrane were removed using a cotton swab. The cells that migrated to the other side of the membrane were fixed in cold methanol for 10 min, stained with 1.4% crystal violet, and washed three times with distilled water. Cell migration was evaluated using photomicrographs from five randomly chosen fields (x100) per insert, and the number of migrated cells was counted using ImageJ 1.45S software as previously described [23]. The number of migrated cells was compared between groups.

### 2.5. RNA Expression Analysis Using Quantitative Real-Time Reverse Transcription Polymerase Chain Reaction (qRT-PCR)

Quantitative real-time RT-PCR was performed to analyze the quantitative expression of genes involved in extracellular matrix production. Confluent cells (10,000 cells/well in a 96-well plate) of hGFs were isolated after rinsing with 0, 10, 20, or 50 *µ*g/ml vitamin C in serum-free medium for 7 min and repeated 3 times a day. Total mRNA was isolated using trizol reagent (#2302700, Prime, Gaithersburg, MD, USA). The lysate was extracted by adding 100 *µ*l chloroform, mixing, and centrifuging for 15 min at 12,000 rpm. The mRNA was precipitated with 250 *µ*l isopropanol, and the pellets were dissolved in nuclease-free water. The amount of RNA was measured using a spectrophotometer (NanoDrop2000, Thermo Scientific, Wilmington, DE, USA). First-strand cDNA was synthesized using reverse transcriptase reaction by ImProm-II Reverse Transcription System (#A3800, Promega Corporation, Madison, WI, USA), and quantitative PCR was performed following the manufacturer's instructions.

PCR primer for type I collagen (COL1), focal adhesion kinase (FAK), fibronectin (FN), interleukin-6 (IL-6G), basic fibroblast growth factor (bFGF), and vascular endothelial growth factor (VEGF) (Table 1) were used to screen extracellular matrix gene expression. All graphs were analyzed and normalized with the expression of the reference gene; Then, the data were analyzed using Bio-1D software version 15.03 (Vilber Lourmat, Marne La Vallée, France). Three independent experiments were repeated for each donor line to compare the gene expression fold-change.

### 2.6. Data Analysis

All experiments were repeated three times. The data are shown as the means and standard deviations (SD) for the remaining wound area, cell viability, cell migration, and gene expression fold-change. The SPSS v.21 (IBM, New York, NY, USA) program was used for statistical analysis. Data normality was evaluated using the Kolmogorov–Smirnov test. The Kruskal–Wallis test was used to compare the results between groups at a significance level of 0.05.

## 3. Results

**Figure 1:**
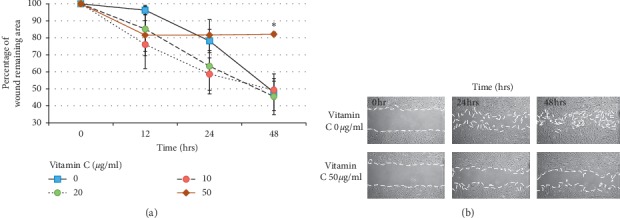
Scratch test assay. (a) Remaining wound area in scratch test assay at 48 h. Rinsing with 50 *µ*g/ml vitamin C significantly delayed wound closure compared with the control group (^*∗*^*p* ≤ 0.05). (b) Scratch test assay images of 50 *µ*g/ml vitamin C and control groups at 0, 24, and 48 h after rinsing with vitamin C.

The results of the scratch assay indicated that rinsing with 10 or 20 *µ*g/ml vitamin C demonstrated no significant effect on in vitro wound closure. However, at 48 hours, 50 *µ*g/ml vitamin C induced significantly delayed wound closure compared with the control group (*p*=0.012). Means of percentage of wound remaining area at 12 hours were 94.49 ± 4.9, 77.92 ± 9.93, 81.25 ± 7.27, and 84.45 ± 9.28 in groups of vitamin C 0, 10, 20, and 50 *µ*g/ml, respectively. Means and standard deviations of percentage of wound remaining area at 24 hours were 72.16 ± 12.32, 52.59 ± 9.94, 68.91 ± 15.23, and 79.25 ± 11.41 in groups of vitamin C 0, 10, 20, and 50 *µ*g/ml, respectively. Means and standard deviations of percentage of wound remaining area at 48 hours were 46.35 ± 9.58, 39.82 ± 8.44, 42.28 ± 8.22, and 78.21 ± 10.98 in groups of vitamin C 0, 10, 20, and 50 *µ*g/ml, respectively (Figure 1).

**Figure 2:**
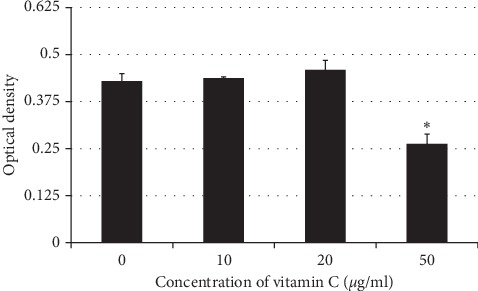
Optical density results of the MTT assay of the 10, 20, and 50 *µ*g/ml vitamin C groups. In a group rinsing with 50 *µ*g/ml vitamin C, it significantly reduced cell viability compared with the control group (^*∗*^*p* ≤ 0.05).

Corresponding with the wound healing assay results, we found that 10 and 20 *µ*g/ml vitamin C did not significantly affect cell viability. In contrast, 50 *µ*g/ml vitamin C significantly reduced cell viability compared with the control group (*p*=0.005). Means and standard deviations of cell viability test was 0.37 ± 0.047, 0.374 ± 0.05, 0.379 ± 0.065, and 0.253 ± 0.02 in groups of vitamin C 0, 10, 20, and 50 *µ*g/ml, respectively (Figure 2).

**Figure 3:**
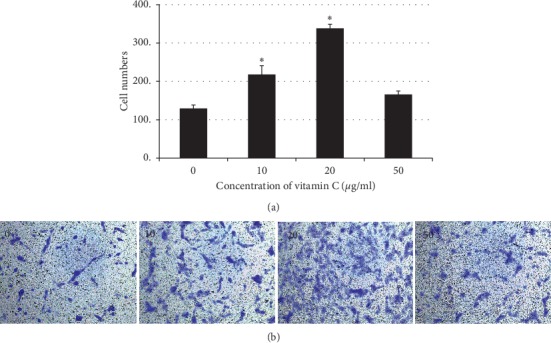
Transwell migration assay. (a) Number of migrated cells in the 10, 20, and 50 *µ*g/ml vitamin C groups. The number of migrated cells was significantly higher after rinsing with vitamin C 10 and 20 *µ*g/ml (^*∗*^*p* ≤ 0.05). (b) Purple spots indicate migrated cells at 24 h.

The transwell assay results revealed that rinsing with 10 or 20 *µ*g/ml vitamin C increased fibroblast migration after 24 h in a concentration-dependent manner (*p*=0.016) (Figure 3). However, the enhanced cell migration observed at the lower vitamin C concentrations was not observed when the dose of vitamin C was increased to 50 *µ*g/ml. Means and standard deviations of transwell-migration assay were 125.25 ± 17.08, 233.98 ± 27.2, 341.52 ± 21.09, and 160.3 ± 55.17 in groups of vitamin C 0, 10, 20, and 50 *µ*g/ml, respectively.

**Figure 4:**
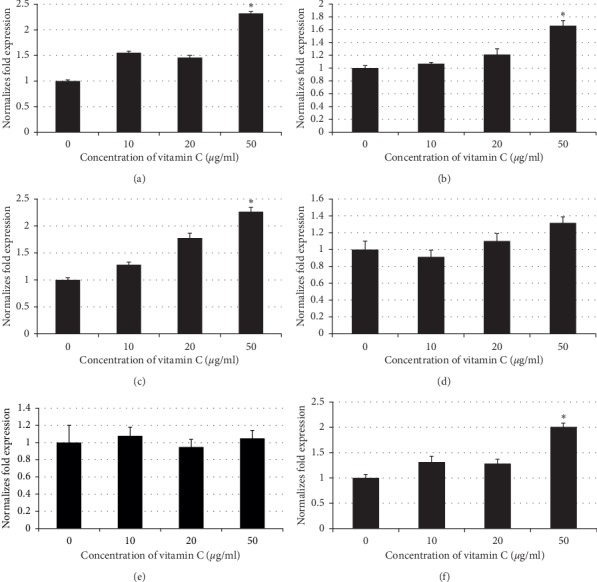
mRNA expression in hGFs rinsed with 10, 20, or 50 *µ*g/ml vitamin C. (a) Collagen I, (b) fibronectin, (c) interleukin-6, (d) focal adhesion kinase, (e) vascular endothelial growth factor, and (f) basic fibroblast growth factor. Rinsing with 50 *µ*g/ml vitamin C significantly upregulated the expression of COL1, fibronectin, IL-6, and bFGF compared with the control group (^*∗*^*p* ≤ 0.05).

We used real-time RT-PCR to analyze the expression of wound healing-related genes in gingival fibroblasts after rinsing with 10, 20, or 50 *µ*g/ml vitamin C. The results indicated that rinsing hGFs with 50 *µ*g/ml vitamin C significantly upregulated the expression of COL1, fibronectin, IL-6, and bFGF compared with the control group (*p*=0.003, 0.005, 0.002, and 0.002, respectively). No significant differences in FAK or VEGF expression were observed between the groups. Means and standard deviations of collagen expression were 0.98 ± 0.16, 1.55 ± 0.13, 1.45 ± 0.15, and 2.32 ± 0.14 in groups of vitamin C 0, 10, 20, and 50 *µ*g/ml, respectively. Means and standard deviations of fibronectin expression were 0.88 ± 0.12, 1.07 ± 0.07, 1.21 ± 0.2, and 1.66 ± 0.17 in groups of vitamin C 0, 10, 20, and 50 *µ*g/ml, respectively. Means and standard deviations of IL-6 expression were 1.03 ± 0.12, 1.28 ± 0.15, 1.77 ± 0.18, and 2.26 ± 0.17 in groups of vitamin C 0, 10, 20, and 50 *µ*g/ml, respectively. Means and standard deviations of FAK expression were 1.01 ± 0.26, 0.91 ± 0.20, 1.10 ± 0.27, and 1.32 ± 0.23 in groups of vitamin C 0, 10, 20, and 50 *µ*g/ml, respectively. Means and standard deviations of VEGF expression were 0.73 ± 0.28, 1.08 ± 0.20, 0.95 ± 0.15, and 1.05 ± 0.18 in groups of vitamin C 0, 10, 20, and 50 *µ*g/ml, respectively. Means and standard deviations of bFGF expression were 0.85 ± 0.17, 1.32 ± 0.58, 1.28 ± 0.24, and 2.01 ± 0.20 in groups of vitamin C 0, 10, 20, and 50 *µ*g/ml, respectively (Figure 4).

## 4. Discussion

The present study demonstrated that rinsing with 10, 20, or 50 *µ*g/ml vitamin C differentially affected the viability, migration, and extracellular matrix protein expression behavior of human gingival fibroblast. Low doses of vitamin C (10, 20 *µ*g/ml) significantly induced fibroblast migration, an early wound healing event, although there was no significance difference in wound closure. In contrast, a higher dose of vitamin C (50 *µ*g/ml) delayed gingival wound closure by inhibiting gingival fibroblast viability. The real-time PCR results demonstrated that 50 *µ*g/ml vitamin C promoted the production of the extracellular matrix proteins, collagen and fibronectin, as well as bFGF and the inflammation mediator, IL-6.

Rinsing with vitamin C at the lower doses enhanced gingival fibroblast migration as shown in the transwell migration assay. This effect was not found at the higher dose (50 *µ*g/ml) of vitamin C we evaluated. Rinsing with 50 *µ*g/ml vitamin C significantly reduced cell viability. Thus, 50 *µ*g/ml vitamin C may have resulted in delayed gingival wound healing in our model due to the reduced cell viability at this concentration. However, 50 *µ*g/ml vitamin C promoted ECM protein expression. This concentration may induce a switch in hGF activity from cell viability and migration to ECM production. Our findings revealed that the effects of vitamin C were dose-dependent corresponding with the findings of Rahman et al. [24].

The higher concentrations of vitamin C, resulting in reduced cell viability, are unresolved. Higher concentrations of vitamin C may result in higher medium acidity, which might be toxic to the cells. However, we measured the pH of each vitamin C concentration in the cell culture medium and found no difference in pH; thus, we can exclude the effect of acidity in our model. Similar to our results, studies demonstrated that vitamin C reduced the proliferation of many cancer cells by unknown mechanisms; however, apoptosis might be involved [25, 26]. The effect of vitamin C on inducing apoptosis is, therefore, an interesting topic that should be explored in future studies. Based on our results, a higher concentration of vitamin C may result in fibroblast production of ECM; however, lower concentrations of vitamin C induced increased proliferation and migration.

Abrahmsohn et al. demonstrated that vitamin C accelerated extraction socket healing and reduced postoperative complications, such as dry socket [18]. They recommended prescribing vitamin C to patients after oral surgery. These authors proposed that the systemic effect of vitamin C on wound healing may be regulated by various pathways, which requires further investigation. The current study regarding the effect of vitamin C on regulating the in vitro wound healing activity of gingival fibroblasts is the first report of the local effect of vitamin C on wound healing. The design of our study intended to mimic using vitamin C in the form of a mouth rinse or oral lozenge that slowly dissolves in the mouth. We aimed to use oral lozenge dissolved as a rinsing effect in patients who undergo oral surgery to accelerate wound healing in our future study. Therefore, we calculated mean duration of vitamin C 500 mg oral lozenge dissolution from 6 volunteers (6.87 ± 0.31 minutes) and approximately used 7 minutes in our study for rinsing protocol. Hence, our study was designed to mimic this condition by rinsing the cells with various concentrations of vitamin C for 7 min, 3 times/day. Further clinical study is required to investigate if local or rinsing vitamin C in dose-dependent administration promotes better wound healing in extraction socket which may improve a postoperative wound healing in compromised patients or accelerates wound healing for further dental substitution or for esthetic reconstruction.

Vitamin C promotes collagen synthesis. Mohammed BM et al. demonstrated that administering vitamin C to mice increased collagen synthesis in the wound compared with the control group [1]. Tsutsumi et al. demonstrated that L-ascorbic acid 2-phosphate magnesium salt (a long-lasting L-ascorbic acid) was more effective in inducing collagen I gene expression compared with normal L-ascorbic acid [12]. Their results are consistent with those of our study.

Epithelial and fibroblast cells secrete IL-6 to activate the inflammatory phase of wound healing [27]. IL-6 also promotes epithelium growth during the proliferation phase [4]. In our study, we found increased IL-6 expression after rinsing the hGFs with vitamin C. This effect might be beneficial for promoting wound healing *in vivo*.

Numerous studies have investigated the effect of vitamin C to treat various types of cancer [13, 25, 28]. The results of our study extend the basic knowledge of the effect of vitamin C on gingival fibroblast behavior, and we propose vitamin C as a supplement that has the potential to be used orally to promote oral wound healing. Further clinical studies should be performed to validate our in vitro findings.

## 5. Conclusions

Rinsing with lower doses of vitamin C significantly induces fibroblast migration; however, they do not affect *in vitro* gingival wound closure. Moreover, a higher dose of vitamin C delays wound closure by inhibiting gingival fibroblast viability. In contrast, higher dose of vitamin C promotes ECM protein and inflammatory cytokine production. Vitamin C is safe and can be prescribed to patients after oral surgery; however, the suitable dosage and methods of administration should be determined to maximize its benefit.

## Figures and Tables

**Table 1:** Primers' sequences.

Name	Sequence (5′–3′)
COL1-FP	GCA AAG AAG GCG GCA AA
COL1-RP	CTC ACC ACG ATC ACC ACT CT
FAK-FP	CAA TCC CAC ACA TCT TGC TGA
FAK-RP	AGC CGG CAG TAC CCA TCT ATT
FN-FP	GGA TCA CTT ACG GAG AAA CAG
FN-RP	GAC ACT AAC CAC ATA CTC CAC
IL-6-FP	GGA TTC AAT GAG GAG ACT TGC C
IL-6-RP	TCT GCA GGA ACT GGA TCA GG
bFGF-FP	GGC TTC TTC CTG CGC ATC CAC
bFGF-RP	GGT AAC GGT TAG CAC ACA CTC CT
VEGF-FP	ATG AGG ACA CCG GCT CTG ACC A
VEGF-RP	AGG CTC CTG AAT CTT CCA GGC A
GAPDH-FP	TGA AGG TCG GAG TCA ACG GAT
GAPDH-RP	TCA CAC CCA TGA CGA ACA TGG

FP: forward primer; RP: reverse primer.

## Data Availability

The data used to support the findings of this study are included within the article.
